# Fusion of Inertial/Magnetic Sensor Measurements and Map Information for Pedestrian Tracking

**DOI:** 10.3390/s17020340

**Published:** 2017-02-10

**Authors:** Shu-Di Bao, Xiao-Li Meng, Wendong Xiao, Zhi-Qiang Zhang

**Affiliations:** 1 School of Electronic and Information Engineering, Ningbo University of Technology, Ningbo 315200, China; shudi.bao@nbut.edu.cn; 2 Institute for Infocomm Research, Singapore 138632, Singapore; xlmeng@i2r.a-star.edu.sg; 3 School of Automation and Electrical Engineering, University of Science and Technology Beijing, Beijing 100083, China; wdxiao@ustb.edu.cn; 4 School of Electronic and Electrical Engineering, School of Mechanical Engineering, University of Leeds, Leeds LS2 9JT, UK

**Keywords:** sensor fusion, gait analysis, bio-motion analysis, rehabilitation, zero velocity update, map information, particle filter

## Abstract

The wearable inertial/magnetic sensor based human motion analysis plays an important role in many biomedical applications, such as physical therapy, gait analysis and rehabilitation. One of the main challenges for the lower body bio-motion analysis is how to reliably provide position estimations of human subject during walking. In this paper, we propose a particle filter based human position estimation method using a foot-mounted inertial and magnetic sensor module, which not only uses the traditional zero velocity update (ZUPT), but also applies map information to further correct the acceleration double integration drift and thus improve estimation accuracy. In the proposed method, a simple stance phase detector is designed to identify the stance phase of a gait cycle based on gyroscope measurements. For the non-stance phase during a gait cycle, an acceleration control variable derived from ZUPT information is introduced in the process model, while vector map information is taken as binary pseudo-measurements to further enhance position estimation accuracy and reduce uncertainty of walking trajectories. A particle filter is then designed to fuse ZUPT information and binary pseudo-measurements together. The proposed human position estimation method has been evaluated with closed-loop walking experiments in indoor and outdoor environments. Results of comparison study have illustrated the effectiveness of the proposed method for application scenarios with useful map information.

## 1. Introduction

Wearable inertial/magnetic sensor based human lower body motion analysis has been widely applied in a variety of applications, such as animation, entertainment, sports training, gait analysis and rehabilitation [1,2,3]. For the lower body bio-motion analysis, extensive research has been performed to make estimated motions visually similar to real human subject movements [4,5], but how to reliably provide position estimation of the human subject during walking still remains challenging.

Human position estimation normally requires three pre-installed access points or beacon nodes at any point in the service area. A typical example of this type of system is the global positioning system (GPS), which is commonly used in outdoor environments. However, due to the GPS signal attenuation caused by buildings, tunnels, and other construction materials, GPS may not be applicable for robust and accurate indoor human position estimation [6,7]. Alterative solutions, such as WiFi and Bluetooth beacons, magnetometer, vision, or ultrasound for indoor localization have also been explored so far [8,9], but such systems require extensive setup and calibration of the tracking volume, which may be of limited size and may suffer from occlusion.

For this purpose, inertial/magnetic measurements have been explored for human position tracking in arbitrary unprepared indoor and outdoor environments. Thus far, two main approaches for human position estimation using inertial/magnetic sensors are identified [10]. One method is to make use of walk dynamics to determine positions by aggregating individual steps, with some researchers reporting good results [11,12].

The second approach is based on the well-known strap-down navigation algorithm, and the work described here belongs to this latter approach. The basic idea of the strap-down navigation algorithm for position estimation is to (1) acquire the human motion-induced linear acceleration by removing the gravitational acceleration from accelerometer measurements; and (2) integrate the derived linear acceleration twice to estimate positions. Although extensive research has been performed on how to fuse inertial/magnetic sensor measurements for accurate attitude estimation and linear acceleration extraction [13,14], it is still extremely difficult to extract accurate linear accelerations from accelerometer signals due to sensor bias and noises. However, there are repeated recognizable stance phases in gait cycles, during which the foot stays stationary on the ground to support the other leg swing forward and both the velocity and acceleration of the foot are zero. Such a constraint, called zero velocity update (ZUPT) has been widely applied to reset the accumulated errors in the double linear acceleration integration process. Thus far, different types of ZUPT methods have been proposed for human position estimation. For instance, Foxlin [15] and Godha et al. [16] proposed to reset the integrated velocity to zero directly during the stance phases by introducing ZUPT as pseudo-measurements into an extended Kalman filter. However, such methods only used ZUPT information in stance phases, and ignored accumulated errors in non-stance phases of gait cycles. To overcome the issue of simply resetting the accumulated velocity error periodically, Yun et al. [17] further improved the idea of ZUPT and introduced a time variant acceleration bias error to revise the acceleration in the non-stance phases. Although the removal of the acceleration bias error can significantly improve the accuracy of position tracking, it is still problematic for long distance tracking. Similarly, both Schepers et al. [18] and Floor-Westerdijk et al. [19] proposed to use high pass filters to remove the integration drift. The integrated velocity and the integrated position were high-pass filtered by first-order recursive Butterworth filters, but it is quite challenging to determine the cutoff frequencies of the filters, which makes this method not straightforward to use in practice.

The motivation of the paper is to further extend the method presented in [17] and tackle human position estimation over long duration and distance. Although the incorporation of extra infrastructures, such as ultrasound, short-range radio (Wi-Fi, Ultra-Wideband (UWB), radio frequency identification (RFID) and Zigbee) or vision [20,21], can improve the tracking accuracy for long distance scenarios, the extra instrumentations make the systems less ubiquitous in terms of installation and maintenance. However, instead of using extra infrastructures, other information, such as map information can also be acquired in advance and used for human position estimation [22]. Therefore in this paper, we integrate the ZUPT and map information together in the framework of particle filter to further correct the acceleration double integration drift and thus improve the tracking accuracy. The main contributions of this paper include: (1) we design a simple and reliable method of key gait events detection to identify the stance phase of a gait cycle based on the gyroscope measurements; (2) in the framework of Bayesian dynamics, we introduce an acceleration control variable derived from ZUPT in the process model for the non-stance phases, while the map information is taken as the binary pseudo-measurement to further reduce uncertainty of walking trajectories; (3) a particle filter is then designed to fuse ZUPT information and binary pseudo-measurements together. The proposed human position estimation method has been extensively evaluated with indoor and outdoor walking experiments, and results of comparison study have illustrated the effectiveness of the proposed method for application scenarios with useful map information.

The rest of the paper is organized as follows. Section II presents the proposed position estimation method under the framework of the particle filter, including the gait events detection, motion-induced linear acceleration derivation, process model, measurement model and particle filtering. Experimental results and discussions are described in Section III. Finally, we conclude the work in Section IV.

## 2. Proposed Position Estimation Method

**Figure 1:**
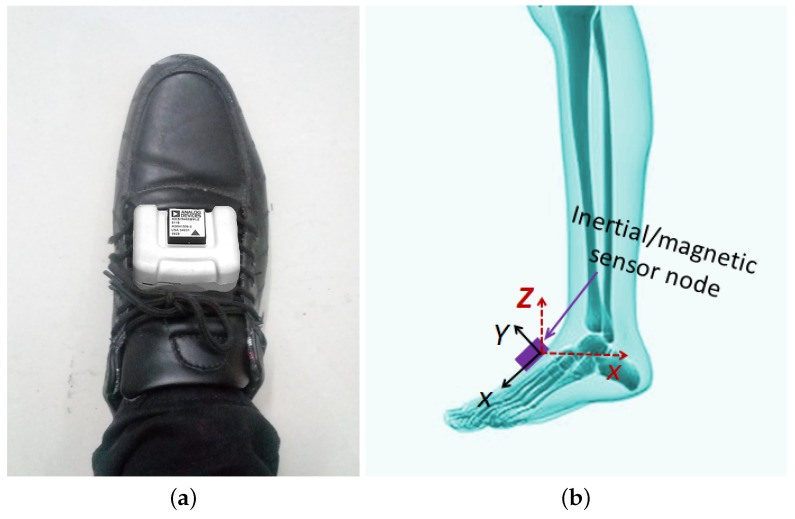
The attachment of a sensor module on the foot of a subject and the illustration of the coordinate systems. The body coordinate system is given in red dashed lines, while the sensor coordinate system is given in black solid lines.

Before we start to introduce the proposed position estimation method, three coordinate systems are defined as follows. (1) The global coordinate system, i.e., the reference coordinate system which will remain unchanged during each trial; (2) Body coordinate system, i.e., the coordinate system of foot segment, *X* axis pointing the backward direction of the subject, *Y* axis pointing right and *Z* axis pointing up. As shown in Figure 1, a sensor module was put on the foot of a subject, and the global coordinate system and body coordinate system coincide with each other at the initial position of each trial. Please be noted that the sensor can only be placed on the foot in order to make use of ZUPT information; (3) Sensor coordinate system, i.e., three orthogonally axes of the mounted sensors. To facilitate our analysis, the body coordinate system is assumed to coincide with the sensor coordinate system after sensor to body alignment calibration, and the world coordinate system is selected to coincide with the body coordinate system at its initial standing position in our implementation [23].

**Figure 2:**
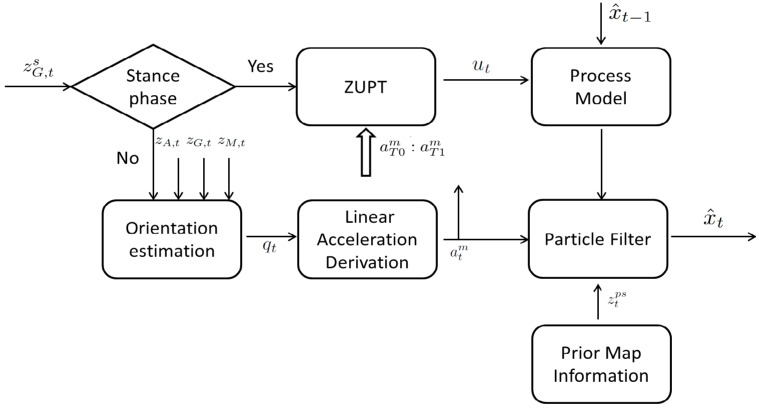
Flowchart of the proposed position tracking algorithm.

Figure 2 depicts the work flow of the proposed position estimation method. Once a stance phase is detected, it will fuse ZUPT and map information using a particle filter to estimate the pedestrian position for the previous non-stance phase. Detailed descriptions of each part of the method is given below.

### 2.1. Stance Phase Detection

**Figure 3:**
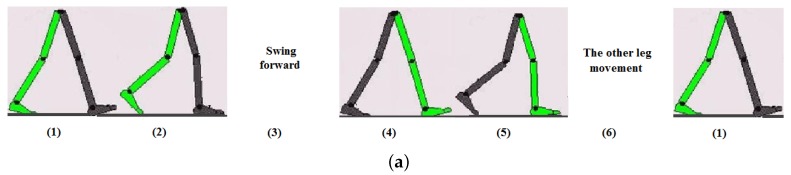

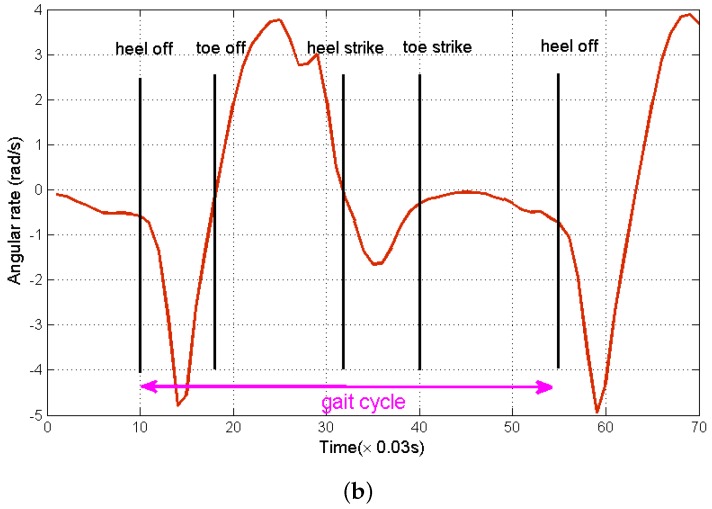
Illustration of the lower body movement during a gait cycle. (**a**) Take the leg in green for example, a gait cycle mainly consists of 6 steps, i.e., (1) the heel leaves the ground (heel off), (2) the toe leaves ground (toe-off), (3) the leg swings forward, (4) the heel contacts the ground (heel-strike), (5) the toe contacts the ground (toe-strike), (6) the stance phase when foot stays on the ground to support the other leg swing forward; (**b**) The gyroscope signal in the sagittal plane during a gait cycle, where the gyroscope signal is close to 0 and the variations are subtle during the stance phase.

The gait cycle depicted in Figure 3 is used to describe the complex activity of walking, which includes the motions from initial supporting heel leaving the ground to the same heel leaving the ground for a second of time [24]. As can see from the figure, the gait cycle mainly consists of 6 stages, and only when the foot stays on the ground to support the other leg swing forward, the gyroscope signal is close to 0 and the variations are subtle. Since the zero-velocity constraint only exists during the stance phase of a gait cycle, we will only detect this phase which starts from the toe strike and ends at heel off.

According to the characteristics of gyroscope measurements in the sagittal plane, a simple stance phase detector can be designed as:

(1) $$\mathrm{Stance}=\left\{\;\begin{array}{c} 1,\;\mathrm{if}\;∥z_{G,t}^{s}∥<\lambda_{1}\mathrm{and}∥\frac{z_{G,t}^{s}-z_{G,t-1}^{s}}{\delta t}∥<\lambda_{2}\\ 0,\;\mathrm{otherwise} \end{array}\right.$$

where $z_{G,t}^{s}$ is the gyroscope reading in the sagittal plane at time *t*, $\delta_{t}$ is the sampling interval (which was set to 0.01 s in our implementation), $\lambda_{1}$ and $\lambda_{2}$ are the predefined positive thresholds which are set empirically to 0.5 rad/s and 0.25 rad/s^2^, respectively. 

### 2.2. Motion-Induced Linear Acceleration Derivation

The accelerometer measurements not only include the gravitational acceleration, but also contain the motion-induced linear acceleration. To estimate the position, the first step is to remove the gravitational acceleration from the accelerometer measurement vector $z_{A,t}$. Since accelerometer readings are given in the sensor coordinate system, transformation of the readings into the global coordinate system is required as

(2) $$a_{t}^{e}=q_{t}⊗z_{A,t}⊗q_{t}^{-1}$$

where $q_{t}$ is the quaternion representing the orientation of the foot, $q_{t}^{-1}$ is the quaternion conjugate, and ⊗ is the quaternion multiplication. There have been a number of sensor fusion methods to estimate orientation $q_{t}$ from the inertial/magnetic sensor measurements [25,26]. In our implementation, we used the algorithm proposed in [26] to estimate the orientation. During the quaternion multiplication in Equation (2), the acceleration vector $a_{t}^{e}$ and $z_{A,t}$ are taken as the pure vector quaternion with the scale part being set to 0. Given the gravitational acceleration $g^{e}$ in the global coordinate system, the motion-induced linear acceleration can be calculated as:

(3) $$a_{t}^{m}=a_{t}^{e}-g^{e}.$$

Theoretically, human position can be obtained by integration of the above acceleration vector twice; however, due to the existence of measurement noise and drift in the measured acceleration vector $z_{A,t}$ and errors in the estimated quaternion $q_{t}$, the double integration of $a_{t}^{m}$ would result in unbounded error in position estimation. In the following sections, we will introduce how to incorporate ZUPT and map information to correct the unbounded drift.

### 2.3. ZUPT and Process Model

For any gait cycle, we can define $T_{0}$ as the time step of the heel off, and $T_{1}$ as the time step of the coming toe strike. Thus the foot starts moving at $T_{0}$ while stop moving at $T_{1}$, which means the velocities at $T_{0}$ and $T_{1}$ should both be 0. However, the direct integration of the linear acceleration $\int_{T_{0}}^{T_{1}}a_{t}^{m}$ may not be zero in practice; therefore, we can define a bias error $u_{t}$ as the input for the process model to overcome this issue:

(4) $$u_{t}=\frac{t-T_{0}}{T_{1}-T_{0}}\int_{T_{0}}^{T_{1}}a_{t}^{m}.$$

Since the process model employed by the particle filter governs the dynamic relationship between the states of two successive time steps, we can define the state vector at time step *t* as $x_{t}$, consisting of position $x_{p,t}$, velocity $x_{v,t}$ and motion acceleration $x_{a,t}$, i.e.,

(5) $$x_{t}=\left[\begin{array}{c} x_{p,t}\\ x_{v,t}\\ x_{a,t} \end{array}\right]$$

Therefore, a simple constant acceleration model can be constructed as

(6) $$x_{a,t}=x_{a,t-1}+w_{a,t}$$

where $w_{a,t}$ is a random Gaussian noise with zero mean and covariance matrix $Q_{a}$.

To reduce the acceleration integration drift during the swing phase, we introduce the acceleration bias as a control variable in the velocity dynamic model. Thus, the velocity can be modeled as:

(7) $$x_{v,t}=x_{v,t-1}+\left(x_{a,t}-u_{t}\right)\delta_{t}+w_{v,t}$$

where $w_{v,t}$ is the velocity process noise with covariance matrix $Q_{v}$.

Then the position of the foot is calculated by

(8) $$x_{p,t}=x_{p,t-1}+x_{v,t}\delta_{t}+\frac{1}{2}\left(x_{a,t}-u_{t}\right)\delta_{t}^{2}+w_{p,t}$$

where $w_{p,t}$ is the position process noise with covariance matrix $Q_{p}$.

From Equations (6)–(8), the linear process model can be summarized as:

(9) $$\begin{array}{c} x_{t}\;=\;\left[\begin{array}{ccc} \;I_{3} & \delta_{t}I_{3} & \;\frac{1}{2}\delta_{t}^{2}I_{3}\\ \;0_{3\times 3} & \;I_{3} & \;\delta_{t}I_{3}\\ \;0_{3\times 3} & \;0_{3\times 3} & \;\;c_{a}I_{3} \end{array}\right]\;x_{t-1}+\left[\begin{array}{c} \;-\frac{1}{2}u_{t}\delta_{t}^{2}\\ \;-u_{t}\delta_{t}\\ \;0_{3\times 1} \end{array}\right]\;\;+\;\;\left[\begin{array}{c} \;w_{p,t}\\ \;w_{v,t}\\ \;w_{a,t} \end{array}\right] \end{array}$$

where $I_{3}$ denotes a 3×3 identity matrix, and 0 stands for zero matrix. In this paper, $w_{p,t}$, $w_{v,t}$ and $w_{a,t}$ are assumed to be uncorrelated with each other, thus the process noise covariance matrix $Q_{t}$ will have the following expression, i.e.,

(10) $$Q_{t}=\mathrm{diag}\left(\left[Q_{p},Q_{v},Q_{a}\right]\right).$$

### 2.4. Measurement Model

Since the motion-induced linear acceleration can be calculated as shown in Equation (3), we can construct a straightforward measurement equation as

(11) $$a_{t}^{m}=x_{a,t}+n_{a,t}$$

where $n_{a,t}$ is the sensor measurement noise with zero mean and covariance matrix $\Sigma_{\;a,t}$.

Furthermore, vector map information can also be used for position estimation. For indoor positioning, building plans are very useful information that can be applied to improve the accuracy of location estimation by reducing the uncertainty of walking trajectories. Similarly, for outdoor positioning, the road information can also be applied to provide an enhanced positioning output. Since the vector map data can be acquired in advance, we can therefore construct the following pseudo-measurement equation, i.e.,

(12) $$z_{t}^{ps}=h\left(x_{t}\right)$$

where

(13) $$\;h\left(x_{t}\right)\;\;=\;\;\left\{\;\;\begin{array}{cc} 1,\;\; & \mathrm{if}\;x_{p,t}\;\text{is inside the possible walking area}\\ 0,\;\; & \mathrm{otherwise} \end{array}\right.$$

### 2.5. Particle Filtering

From a Bayesian fusion perspective, the problem of human position estimation is to recursively calculate the conditional probability density function (pdf) $p(x_{t}|y_{T_{0}:t})$ at time *t*, given all the measurements $y_{T_{0}:T_{1}}\;\;=\;\;\left\{y_{t},t=T_{0}\cdots ,T_{1}\right\}$, where $y_{t}=\left[\begin{array}{c} a_{t}^{m}\\ z_{t}^{ps} \end{array}\right]$. Due to nonlinear and non-Gaussian nature of the pseudo measurement equation, we resort to particle filter approximation for sub-optimal Bayesian solution.

Particle filters or Sequential Monte Carlo (SMC) methods are a set of on-line posterior density estimation algorithms that estimate the posterior density of the state-space by directly implementing the Bayesian recursion equations. The idea of Monte Carlo simulation is to approximate the posterior distribution using *N* identical independently distributed particles $x_{t-1}^{i}$ and associated weights $\pi_{t-1}^{i}$, where i=1,2⋯N (*N* was empirically set as 500 in our implementation) [27]. Particle filters have evolved into many different varieties over the past few years. The key issue is the choice of proposal distribution, which can best approximate the target posterior distribution. Here we choose Sequential Importance Re-sampling (SIR) due to its simplicity and effectiveness [28]. With SIR, new particles are generated according to the process model as

(14) $$x_{t}^{i}=p\left(x_{t}|x_{t-1}^{i}\right)$$

while the new importance weights are updated using the measurement likelihood function, i.e.,

(15) $$\begin{array}{cc} \pi_{t}^{i} & \propto p(y_{t}|x_{t}^{i})\\ \sum_{i}^{N}\pi_{t}^{i} & =1 \end{array}$$

Obviously, the new particles generation is straightforward, given the process model is a linear dynamic model with Gaussian noise as shown in the Equation (9). The difficulty of the SIR is to update the weight. Assume the $a_{t}^{m}$ and $z_{t}^{\mathrm{ps}}$ are independent, the fusion process can be formulated as

(16) $$p\left(y_{t}|x_{t}^{i}\right)=p\left(a_{t}^{m}|x_{t}^{i}\right)\cdot p\left(z_{t}^{ps}|x_{t}^{i}\right)$$

where $p(a_{t}^{m}|x_{t}^{i})$ is straightforward, given the measurement is continuous. Here we propose the likelihood function for the binary measurements $z_{t}^{ps}$ as

(17) $$p\left(z_{t}^{ps}|x_{t}^{i}\right)=\left\{\begin{array}{c} 1,\;\mathrm{if}\;h(x_{t}^{i})=1\\ 0,\;\mathrm{if}\;h(x_{t}^{i})=0 \end{array}\right.$$

To prevent the filter from degeneracy phenomenon, systematic re-sampling was adopted in our implementation.

## 3. Experimental Results

### 3.1. Experimental Setup

To evaluate the performance of the proposed position estimation algorithm, an inertial/magnetic sensor module was placed on the foot as shown in Figure 1a in our experiments. The sensor chip is ADIS16405 from Analog Devices, which contains a triaxial accelerometer, triaxial gyroscope and triaxial magnetometer. The sensor module was connected to a base station by serial peripheral interface (SPI) data bus, which controlled the data collection and sent the data to PC for offline processing through Bluetooth. In order to get clean signals, an analog low pass filter with cutoff frequency at 100 Hz was also applied on the printed circuit board (PCB) to remove high-frequency noise before sending out the data.

In our experiments, closed-loop walking patterns were applied, and five healthy subjects (including 3 males and 2 females) were asked to walk in the corridor along the predefined path back to the starting point at their comfortable speed. The subjects’ average age was 32 with a standard derivation of 4, and the average height was 1.71 m with a standard derivation of 0.07. Based on the measurement results, the averaged walking speed is about 0.75 m per second. To facilitate the walking process, distinctive points along the walking path were marked on the floor to guide the subjects to walk along the path. The corridor information was acquired in advance as the priori map information. To further evaluate the performance of the proposed method, closed-loop outdoor walking experiments were also performed. One trial per subject was performed for each walking pattern for statistic analysis.

### 3.2. Experimental Results and Discussion

A comparison study between the state-of-the-art ZUPT-based methods and our method was carried out to evaluate the position estimation performance. In our experiments, three ZUPT-based methods were implemented, including Godha’s method [16] which simply resets the velocity to zero during the stance phases; Yun’s method [17] which applies a time-variant acceleration bias error to remove velocity drift; and Schepers’ method [18,19] which uses high pass filters to remove velocity drift. To make the comparison fairer, we also implemented the Leppakoski’s map based position estimation method [22]. In what follows, “Truth” represents the marked trajectory that the subjects need to follow, “Our” shows the tracking results of our method, “Lepp” indicates the performance of Leppakoski’s method, and “Godha”, “Yun” and “Schepers” stand for the corresponding ZUPT-based methods, respectively.

**Figure 4:**
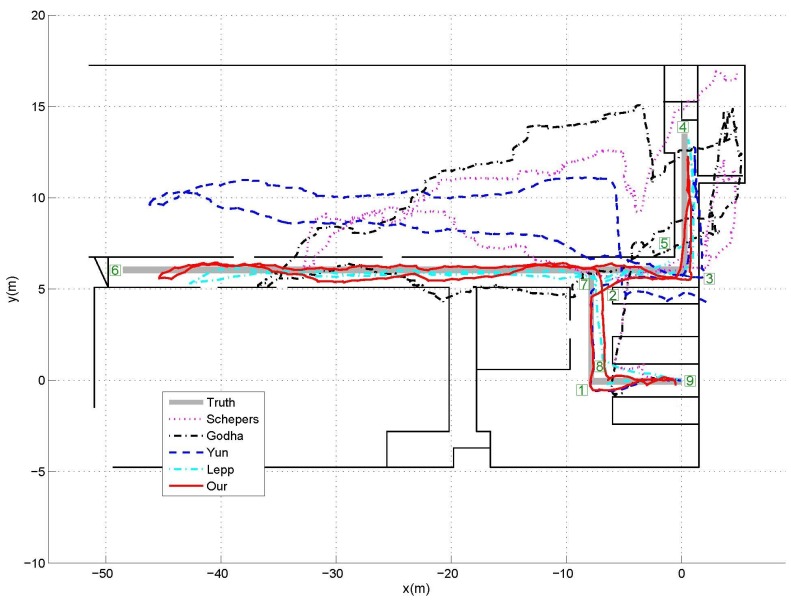
One example of the position estimation results for indoor experiments. Total walking distance was 132 m, at a speed about 0.75 m/s. The actual positions of the 9 critical points were marked by the square number plates. It is obvious that the integration of ZUPT and map information can improve the position estimation accuracy significantly.

The indoor experiments were carried out in the laboratory corridors, as shown in Figure 4, where the predefined walking path was indicated by the thick solid lines. From the starting point (0,0) in the plot, the subjects have to walk along the path, make several turns and then walk back to the starting position to close the loop. Figure 4 also gives one example of position estimation result for the indoor corridor walking experiments. As can see from the figure, it is very clear that the proposed method can achieve the smallest position errors compared with the other three traditional ZUPT methods, which means that the integration of ZUPT and map information can improve the position estimation accuracy significantly. Meanwhile, the Lepp’s step counter plus map information based method can also achieve relatively good performance. This is mainly because the map information can improve the accuracy of walking direction estimation; however due to the difficulty of determining the step length precisely, there are also some errors in the walking trajectory estimations. We also noticed that Yun’s method outperformed the other two ZUPT methods. The reason is Godha’s method only simply reset the velocity to zero during the stance phases and the accumulated drift during the non-stance phases is included in the location estimation. Although Schepers’ method applied first-order recursive Butterworth high pass filters to remove the drift caused by the acceleration bias in the integrated velocity, the filters cannot remove the integration drift due to the difficulty of choosing the proper cutoff frequency. Yun’s method applied an acceleration bias variable not only to revise the integrated velocity during the non-stance phases, but also to set the velocity to zero during the stance phases, which can significantly improve the accuracy of position tracking over the other two ZUPT methods. On the other side, Yun’s method starts to drift away from the predefined path around the point (−9,6). However, the subject cannot enter into the other side of the corridor due to the existence of the wall. This priori map information was taken into consideration in our propose method; therefore, the estimate trajectory can be revised according to such information and thus position estimation accuracy can be improved significantly.

**Figure 5:**
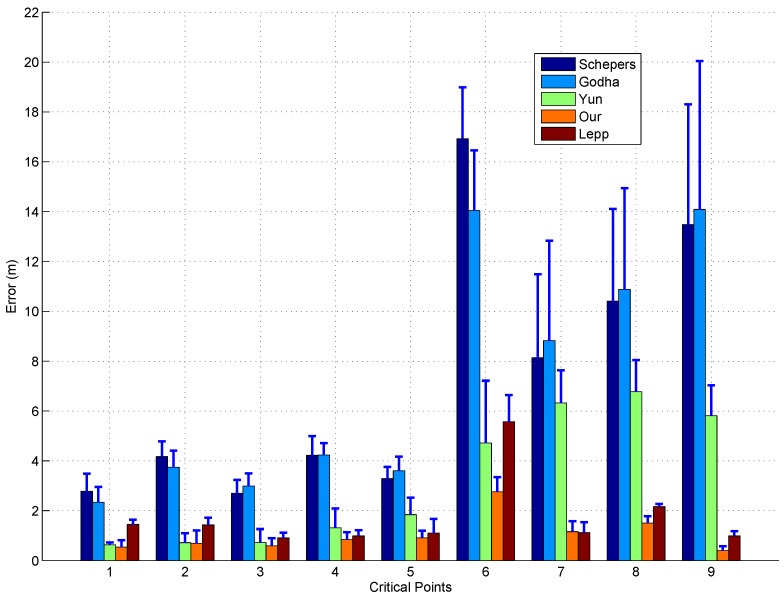
Indoor walking: the average and standard deviation of distance errors between the estimated positions and actual positions of the 9 critical points over 5 trials. For the traditional methods without map information, the distance errors had significant increment as the walking distance increased due to acceleration integration drift.

To further illustrate the strength of the proposed position estimation method, we also compared the estimation results derived from different methods with the predefined path quantitatively. In practice, it is common to assess the estimation accuracy via evaluating the positional difference between the starting and final points for the closed-loop walking patterns [16,17], but such evaluation may not be enough since it ignores the possible deviation of the other points from the walking path. Therefore, as indicated by the square number plates in Figure 4, all the critical turning points including the last point along the predefined path were taken into consideration in our experiment. The locations of these points were manually extracted from the position estimates provided by the four different methods, and then the distance differences between the estimated positions and actual positions of these points were calculated to evaluate the performance of different methods quantitatively. Figure 5 shows the average distance errors and standard deviations between the estimated position and actual position of the 9 critical points from the 5 trials. As can see from the figure, the distance errors have increased significantly as the walking distance increases. This is mainly because the accumulated integration drift will not only affect the current location estimation, but also affect the future location estimation as well. Therefore, once any certain location estimation has a relative large error, the error will pass to the future location estimations and make them also have large errors. Via integration of the priori map information, the accumulated integration drift can be reset, thus even when there is a location estimation with a relative large estimation error, the future location estimation is rarely affected by the error. Taking the critical point 6 for example, for all the five estimation methods, the errors at this point increase significantly over the previous 5 points, which is mainly because that the straight line distance from the original point is the largest. For the three traditional ZUPT methods, the errors at the 3 future points are also very large; however, when the priori map information is taken into consideration in our method and Lepp’s method, the errors at the 3 future points are much smaller than that at the point 6. In summary, integration of the ZUPT-based method with priori map information can achieve much better results than only applying the ZUPT alone. As can also see from the figure, our method surpassed the Lepp’s method slightly. The estimation error over all critical points by our method was 1.03 ± 0.17 m, while that from Lepp’s method was 1.74 ± 0.18 m.

**Figure 6:**
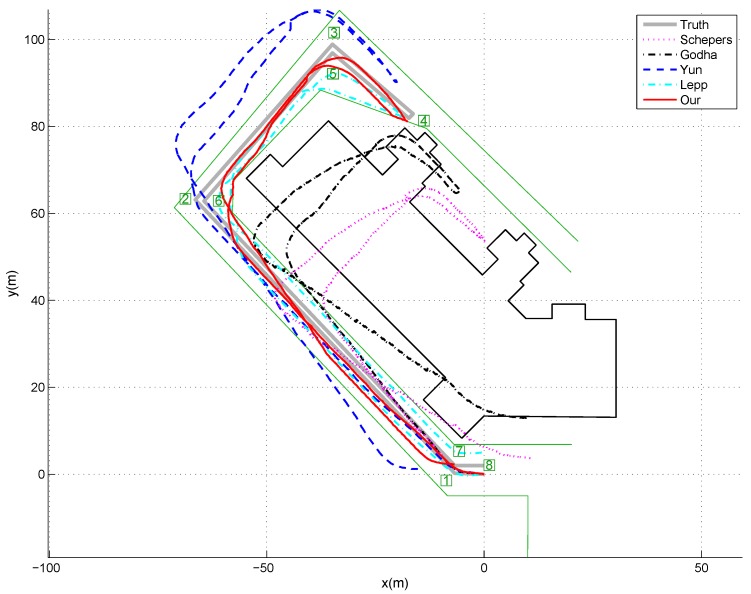
One example of the position estimation for outdoor experiments with a walking speed at about 0.75 m/s. The actual positions of the 8 critical points were marked by the square number plates. It is obvious that the integration of ZUPT and map information can further improve the position estimation accuracy.

**Figure 7:**
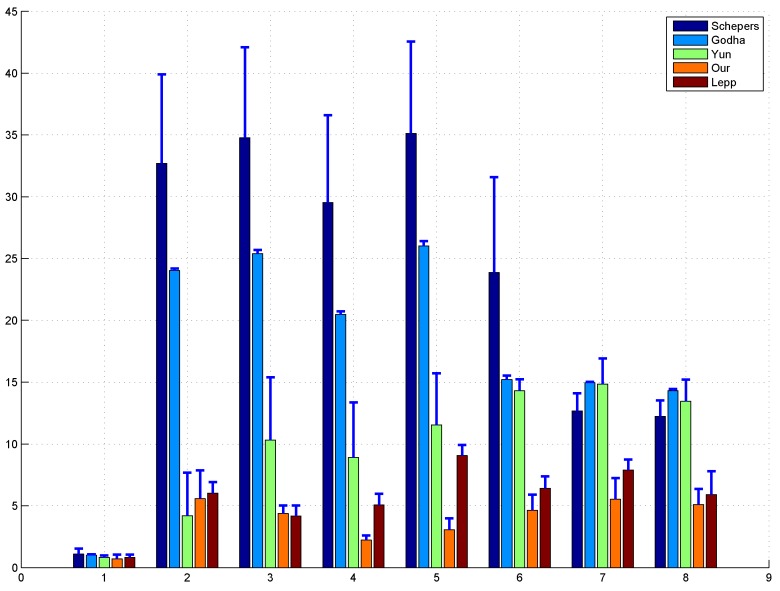
Outdoor walking: the average and standard deviation of distance errors between the estimated positions and actual positions of the 8 critical points over 5 trials. For the traditional methods without map information, the distance errors had significant increment as the walking distance increased due to acceleration integration drift.

Although the outdoor movement can be estimated by GPS easily, we still insist it is worthwhile to evaluate our method in the outdoor environments since GPS signal may not be stable at some points; therefore, some preliminary outdoor closed-loop walking experiments outside our laboratory building were also performed. As shown in Figure 6, the predefined walking path was indicated by the grey solid lines. From the starting position which is the original point in the figure, the subjects walk along the path, make several turns and walk back to the starting position to close the loop. Since our building is surrounded by border hedge and plant, we can assume that the subjects cannot cross the hedge and plant during their walking. On the other side of the road, it is the parking spaces which are normally occupied by vehicles, thus we can also assume the subjects cannot cross the other side either, and they can only walk along the road. Therefore, the road information, given by the light green lines in Figure 6, was acquired in advance and used as the priori map information in our experiments. Similar to the indoor experiments, Figure 6 gives one example of position estimation result for outdoor walking, while Figure 7 shows the average and standard deviation of distance errors between the estimated positions and actual positions of the 8 critical points which were marked by the square number plates in Figure 6 over 5 trials. As can see from these two figures, we can also have (1) the proposed method can achieve the smallest position errors compared with the other three traditional ZUPT methods, which means that the integration of ZUPT and map information can improve the position estimation accuracy significantly; (2) our method also surpassed the Lepp’s method slightly, and the estimation error over all critical points were 3.89 ± 1.10 m and 5.76 ± 0.95 m, respectively, which is mainly due to the existence of inaccurate step length estimation and heading direction error in Lepp’s method; and (3) Yun’s method outperformed the other two ZUPT methods due to the application of a time-variant velocity drift error to revise the acceleration in the non-stance phase. We also noticed that the estimation error of our proposed method has some certain increment for the outdoor walking over that of the indoor walking. The reason for that is the map information used in the outdoor tracking experiment is not as good as the one used for the indoor tracking. In general, the width of a corridor is less than 2 m, while the width of road is larger than 6 m. It means that for the outdoor tracking the map can tolerate much higher errors before resetting the acceleration integration drift; therefore, the accuracy of the proposed method for the outdoor tracking has some slight decrement. Similarly, Lepp’s method also suffered from the degraded heading direction estimation due to the inaccurate map information. On the other side, due to the uncertainty in the step length estimation, the walking trajectory derived from Lepp’s method got significant errors.

**Figure 8:**
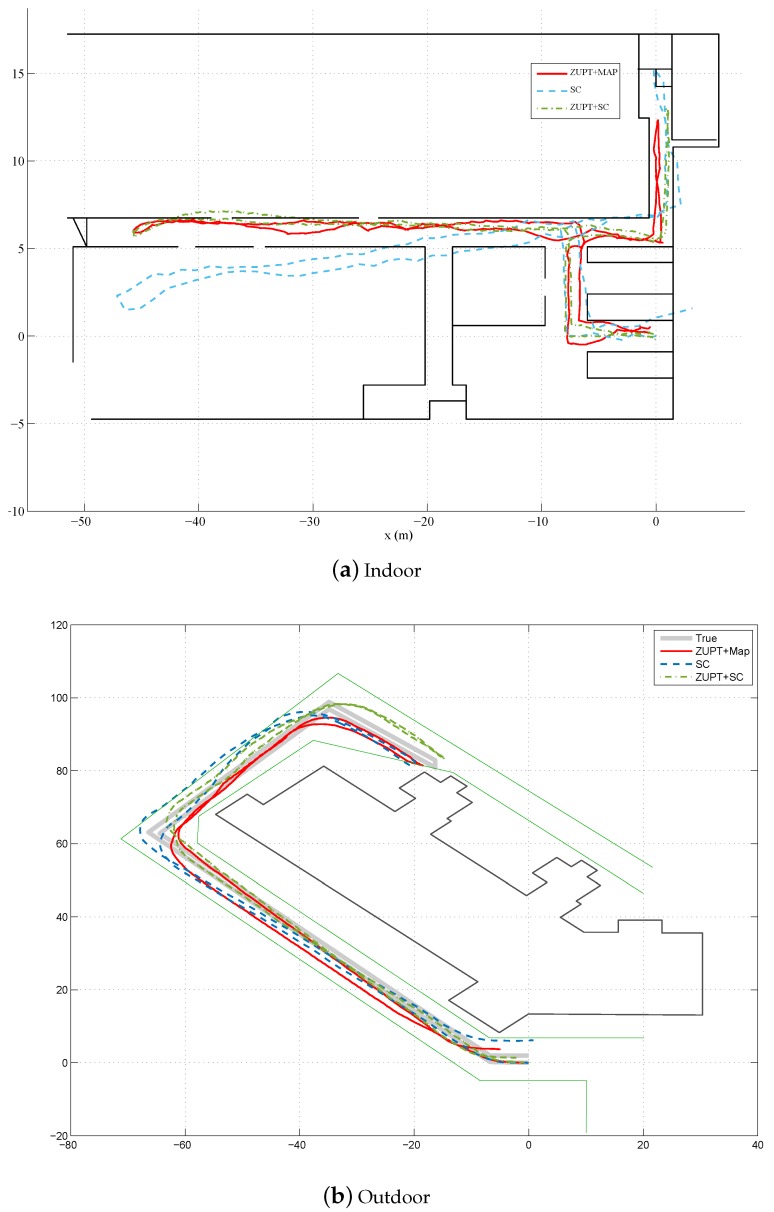
Comparative results with our previous methods presented in [10] and [12]. (**a**) Indoor scenario; and (**b**) Outdoor scenario. For both figures, “ZUPT+SC” is for fusion of ZUPT and step counter presented in [10], “SC” is for the step counter method presented in [12], and “ZUPT+Map” is for fusion of ZUPT and map proposed in this paper.

In our previous works [10,12], we investigated fusion of step counter with ZUPT method for pedestrian location estimation. To demonstrate the pros and cons of these methods, the comparative estimation results of these methods are also given in Figure 8. From both the indoor and outdoor scenarios, we can see that the fusion based methods can outperform the step counter based method, no matter fusing ZUPT with step counter information or fusing ZUPT with map information. Meanwhile, ZUPT+step counter fusion method and ZUPT+map fusion method can achieve quite similar performance. Sometimes, particularly when the map information is very accurate, the ZUPT+map fusion method shows some advantages over ZUPT+step counter fusion method. Take some certain points, such as (−40,7) in the indoor scenario as shown in Figure 8 for example, the estimated trajectory by ZUPT+step counter fusion method may enter into the other side of the corridor. However, this kind of errors can be easily removed by the priori map information. Furthermore, both of our previous works involve stride length estimation, which require to use a specific parameter *K*, while the ZUPT+map fusion method does not need such information. Although such parameter can be determined through offline training by collecting some walking data before the actual experiments from the subject, the value of such parameter may still change for the same person due to some other factors, such as different types of shoes, different sensor sensitivities, different ground surface materials and so on. It means the offline training should be performed frequently before data collections. In contrast, the map information is relatively stable, which does not need to be updated often.

**Figure 9:**
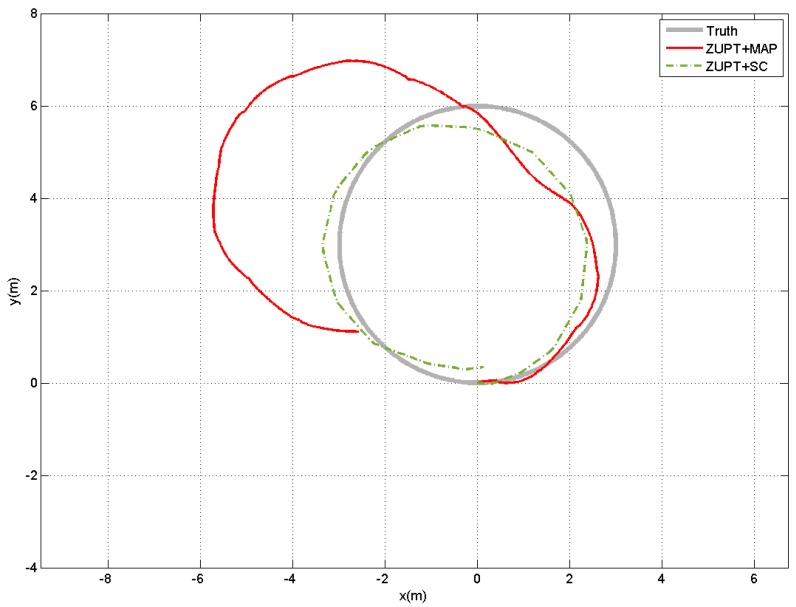
Examples of the estimated position results for walking in a circle with radius of 3 m, where no map information was provided.

When the quality of the map information decreases, the performance of the ZUPT+map information method also declines accordingly. As shown in Figure 8b, the outdoor tracking performance of our method has some slight decrement, while ZUPT+step counter fusion method is more resilient to such variations. However, ZUPT+step counter fusion method may even slightly outperform the ZUPT+map information method, particulary when the map information is not accurate at all. In our experiment, we considered some extreme scenarios when the map information was not available or useable. The subjects were asked to walk inside a big hall where the wall was quite far from the walking region, thus the wall information could not be used for position estimation any more. In this case, the likelihood function for the binary measurements $z_{t}^{ps}$ in Equation (17) will be set to 1 automatically, and the map information is not activated and only the ZUPT information is used in the estimation process to deal with the acceleration double integration drift. Figure 9 shows an example of the estimated displacement results by this method. It is obvious that the less quality the map is, the less contribution of map information to the ZUPT+map method, and the ZUPT+map method would be more equivalent to the ZUPT only method. It is also clear that the ZUPT+step counter fusion method achieves better performance than the ZUPT+map method. Furthermore, the estimation of ZUPT+step approach always falls into the map region, which means that the map actually does not provide extra information. We also notice that the performance of ZUPT+map method varies a lot among trails. For some trials, it can get reasonably good results while the others have significant errors in the estimated position. In contrast, the ZUPT+step counter fusion method can get consistent results for all the 5 trials performed in our experiment. This is mainly because without the map information the accumulated ZUPT errors cannot be removed. Therefore, to reset the acceleration double integration drift and increase the accuracy of the ZUPT-based position estimation methods, extra information, such as vector map information, step counter information, human biomechanical model information and so on, should be incorporated to further reduce the uncertainty of walking trajectories.

Another weakness of the ZUPT+map information method is that it requires to represent the map information in the global coordinate system. In general, the map information usually reflects the structure of a building, and it is normally given in the earth coordinate system. Therefore, it is critical to align the map information in the global coordinate system and make it useable for particle filter. Currently, there is no good method to solve this problem, so in our implementation we manually adjusted the subject initial orientation to minimise such errors.

It is also worth to mention that another source for estimation error is the misalignment between the map coordinate system and the global coordinate system. In our implementation, we implicitly assumed that the map coordinate system was perfectly aligned with the global coordinate system after sensor to body alignment calibration [23]. However, there are some orientation errors between these two coordinate systems. In theory, the determination of such differences between any two coordinate systems can be modelled as a hand-eye calibration problem, and there are many different approaches have been proposed as far [29]. In practice, it is quite challenging to acquire the sensor orientation information in the map coordinate system; therefore, we did not apply the hand-eye calibration but manually adjusted the subject initial orientation to minimise such errors. Although we have tried our best, the map coordinate system might still not be perfectly aligned with the global system in use, which thus could also bring in some estimation errors in our experiments.

In order to get good location estimation results, motion-induced linear acceleration should be derived as accurate as possible by Equation (3), which depends on the performance of orientation estimation algorithm. In our implementation, we applied our previous method proposed in [26]. In general, such a method can provide accurate orientation estimation (less than 3° RMS error). However, there are definitely some errors in the orientation estimation, particularly when the strong linear acceleration interference and the magnetic disturbance last for a long time. The main reason is due to the inevitable gyroscope integration drift. When the interference and the disturbance exist, our method relies on the gyroscope measurement only, and accelerometer and magnetometer measurements are not able to compensate the gyroscope integration drift. Thus, we have to explore different methods, such as ZUPT and the map information, to compensate for the errors existed in the derived motion-induced linear acceleration for good position estimation.

The last thing to discuss is the potential tradeoff between computational complexity and estimation accuracy while applying particle filter. Though it can be run in real-time on off-the-shell micro computers, the number of particles might affect both the position estimation accuracy and the real-time performance in embedded devices. To make the algorithm implementable in low-cost embedded platforms, effects of particle number as well as subject walking speed on the computational complexity and estimation accuracy should be further investigated in the future.

## 4. Conclusions and Future Work

In this paper, we explored the integration of the ZUPT and map information for position estimation during normal walking. A simple and reliable method of key gait events detection was designed to identify the stance phase of a gait cycle based on the gyroscope measurements. For the non-stance phase during a gait cycle, a ZUPT-based acceleration control variable was designed in the process model, while the map information was taken as the binary pseudo-measurements to further improve the accuracy of the position estimation. A particle filter was then designed to fuse all the sensor information and binary pseudo-measurements together. The proposed method has been evaluated with indoor and outdoor walking experiments, and the comparable results have illustrated the effectiveness of the proposed human position method.

As the proposed method was only evaluated for normal walking experiments on level ground, our future work will focus on further extending our method for other walking patterns, like backwards walking, sideways walking and stair climbing. More complicated walking scenarios will be be tested, and effects of different walking speeds will be investigated in the near future.
